# Involvement of microbiota and short-chain fatty acids on non-alcoholic steatohepatitis when induced by feeding a hypercaloric diet rich in saturated fat and fructose

**DOI:** 10.1017/gmb.2022.2

**Published:** 2022-04-08

**Authors:** Iñaki Milton-Laskibar, Laura Judith Marcos-Zambrano, Saioa Gómez-Zorita, Enrique Carrillo de Santa Pau, Alfredo Fernández-Quintela, Jose Alfredo Martínez, María Puy Portillo

**Affiliations:** 1 Precision Nutrition and Cardiometabolic Health Program, IMDEA Food Institute (Madrid Institute for Advanced Studies), Campus of International Excellence (CEI) UAM+CSIC, Spanish National Research Council, Madrid, Spain; 2 CIBEROBN Physiopathology of Obesity and Nutrition, Institute of Health Carlos III (ISCIII), Madrid, Spain; 3 Computational Biology Group, Precision Nutrition and Cancer Research Program, IMDEA Food Institute, Madrid, Spain; 4 Nutrition and Obesity group, Department of Pharmacy and Food Science, Faculty of Pharmacy, Lucio Lascaray Research Center, University of the Basque Country (UPV/EHU), Vitoria-Gasteiz, Spain; 5 BIOARABA Health Research Institute, Vitoria-Gasteiz, Spain

**Keywords:** Non-alcoholic steatohepatitis, rat, microbiota, high-fat high-fructose diet, short-chain fatty acids

## Abstract

Consumption of high-energy-yielding diets, rich in fructose and lipids, is a factor contributing to the current increase in non-alcoholic fatty liver disease prevalence. Gut microbiota composition and short-chain fatty acids (SCFAs) production alterations derived from unhealthy diets are considered putative underlying mechanisms. This study aimed to determine relationships between changes in gut microbiota composition and SCFA levels by comparing rats featuring diet-induced steatohepatitis with control counterparts fed a standard diet. A high-fat high-fructose (HFHF) feeding induced higher body, liver and mesenteric adipose tissue weights, increased liver triglyceride content and serum transaminase, glucose, non-HDL-c and MCP-1 levels. Greater liver malondialdehyde levels and glutathione peroxidase activity were also observed after feeding the hypercaloric diet. Regarding gut microbiota composition, a lowered diversity and increased abundances of bacteria from the *Clostridium* sensu stricto 1, *Blautia*, *Eubacterium coprostanoligenes* group, *Flavonifractor*, and UBA1819 genera were found in rats featuring diet-induced steatohepatitis, as well as higher isobutyric, valeric and isovaleric acids concentrations. These results suggest that hepatic alterations produced by a hypercaloric HFHF diet may be related to changes in overall gut microbiota composition and abundance of specific bacteria. The shift in SCFA levels produced by this unbalanced diet cannot be discarded as potential mediators of the reported hepatic and metabolic alterations.

## Introduction

Obesity is a chronic metabolic disease featuring an excessive body fat accumulation that may impair health status and life expectancy (WHO, 2020). The prevalence of obesity has worldwide exponentially increased in the last decades, becoming one of the most prevalent chronic non-communicable diseases, which is expected to affect more than 1-billion people by the year 2025 (World obesity, 2021). Obesity leads to the development of other pathological conditions and morbid manifestations, such as diabetes, cardiovascular diseases, hypertension, certain types of cancer and non-alcoholic fatty liver disease (NAFLD; Blüher, 2019; Silveira et al., 2021). Indeed, obesity is currently considered as one of the main causes of early deaths in most developed countries (Blüher, 2019).

Sedentary lifestyle habits and/or excessive energy intake are usually the main contributors to the development of this multifactorial disease. Moreover, endogenous factors such as genetic predisposition (mainly single nucleotide polymorphisms) and altered gut microbiota composition are also involved (Hwalla and Jaafar, 2021). In this scenario, the food industry has modified the composition of a wide variety of foods and foodstuffs, exchanging calories coming from fat (the most energy-yielding nutrient) for those coming from different sugars in order to reduce their energy density and thus the energy intake of consumers. Unfortunately, this strategy not only has not been effective in blunting the aforementioned obesity prevalence increase, but it has contributed to the increase in added dietary fructose consumption, which has been related to NAFLD development (Softic et al., 2016). Indeed, once fructose is absorbed in the intestine, the majority of this monosaccharide in the portal vein enters the liver for subsequent metabolic utilization (Hannou et al., 2018). Within the liver, fructose is known to impair hepatic lipid metabolism by enhancing *de novo* lipogenesis, as well as reducing hepatic fatty acid oxidation, which may result in hepatic lipid accumulation (Hannou et al., 2018). Moreover, it must be noted that fructose can also induce further dysfunction in the liver (inflammation, oxidative stress and mitochondrial dysfunction), thus contributing to the onset and progression of NAFLD from relatively benign conditions (hepatic steatosis) towards more harmful ones (steatohepatitis, cirrhosis and hepatocellular carcinoma; Jegatheesan and De Bandt, 2017). Of note, high-fructose intake is also known to induce hepatic insulin resistance due to enhanced fatty acid synthesis and decreased oxidation (resulting in mitochondrial dysfunction), increased reactive oxygen species (ROS) production and/or endoplasmic reticulum stress induction (Softic et al., 2019; Wang et al., 2015).

Moreover, the relationship between fructose and NAFLD is not restricted to the effects exerted by the sugar in the liver (Jegatheesan et al., 2016). Indeed, gut microbiota composition and permeability alterations have been reported in trials conducted in rodents fed diets rich in fructose (Jegatheesan et al., 2016). In this context, the “multiple hit” theory, which is currently used to describe NAFLD development, considers gut microbiota alteration as one of the potential mechanisms underlying this morbid liver condition (Buzzetti et al., 2016). In addition, the alterations induced by fructose in gut microbiota composition can also impair the metabolite profile produced by intestinal bacteria (Buzzetti et al., 2016). Among them, much attention has been paid to short-chain fatty acids (SCFAs) due to their effects on energy metabolism or immune response, as well as their ability to act as signalling molecules (Chakraborti, 2015). These lipid species are the product of indigestible carbohydrate fermentation by intestinal bacteria (Aragonès et al., 2019), and when gut microbiota dysbiosis occurs, their concentration may be impaired, affecting hepatic lipid metabolism (Alves-Bezerra and Cohen, 2017).

In this scenario, the aim of this study was to determine the relationship between changes in gut microbiota composition and SCFA levels induced by a high-fat, high-fructose (HFHF) feeding in rats. Likewise, the role played by SCFAs in the development of steatohepatitis was also analysed.

## Material and methods

### Animals, diets and experimental design

This study was conducted using 20 six-week-old male Wistar rats (Envigo, Barcelona, Spain), and all the experimental procedures were carried out in agreement with the Ethical Committee of the University of the Basque Country (document reference CUEID CEBA/30/2010), according to the European regulations (European Convention-Strasburg 1986, Directive 2003/65/EC and Recommendation 2007/526/EC).

Rats were housed in polycarbonate metabolic cages (Tecniplast Gazzada, Buguggiate, Italy) in an air-conditioned room (22°C) with a 12-hour light/dark cycle. After a 6-day adaptation period, the animals were randomly distributed into two groups of ten animals each: the control group, in which animals were fed a standard diet (AIN-93G, OpenSource Diets, Denmark, D10012G) and the HFHF group, in which animals were fed an HFHF diet (OpenSource Diets, Denmark, D09100301; Supplementary Table S1). These experimental conditions were maintained for 8 weeks and animals had free access to food and water throughout this time frame. Once the whole experimental period was completed, animals were sacrificed after overnight fasting under anaesthesia (chloral hydrate) by cardiac exsanguination.

Body weight and food intake were monitored daily. Faecal samples were collected and processed as explained elsewhere (Milton-Laskibar et al., 2021). Serum was obtained by blood sample centrifugation after clotting (1,000 g for 10 minutes, at 4ºC). Liver, as well as different white adipose tissue depots (subcutaneous, epididymal, perirenal and mesenteric) were dissected, weighed and immediately frozen in liquid nitrogen. Fresh faecal samples were collected at the end of the intervention period, prior to the overnight fasting. To do so, the animals were taken one at a time and housed in a clean, single cage to separately obtain faeces directly after defecation induced by a soft abdominal massage. All samples were stored at –80ºC until analysis.

### Determination of liver triacylglycerol content and blood markers

Total liver lipids were extracted following the method described by Folch et al. (1957), dissolved in isopropanol and subsequently measured using a commercial spectrophotometric kit (SpinReact, Girona, Spain). Commercially available spectrophotometric kits were also used to determine serum glucose (Biosystems, Barcelona, Spain), alanine aminotransferase (ALT) and aspartate aminotransferase (AST) levels. An enzyme-linked immunosorbent assay kit was used to measure serum monocyte chemoattractant protein-1 (MCP-1; Abyntek, Derio, Spain) levels.

### Hepatic oxidative stress markers

A commercial thiobarbituric acid reactive substances (TBARSs) assay kit (Cayman Chemical¸ Ann Arbor, MI, USA) was used to analyse lipid peroxidation in rat liver lysates. The malondialdehyde (MDA) and TBARS adduct resulting from their reaction in an acid medium was measured using an Infinite 200Pro plate reader (Tecan, Männedorf, Zürich, Switzerland). The obtained results were expressed as μg MDA/mg of tissue.

The activity of catalase (CAT) was studied as described elsewhere (Gómez-Zorita et al., 2020) following the method described by Aebi (1984), measuring the H_2_O_2_ disappearance spectrophotometrically at a wavelength of 240 nm. Catalase activity was expressed as nmol/min/μg of protein. Glutathione peroxidase (GPx) activity was measured spectrophotometrically in liver lysates using a commercial kit (Biovision, Milpitas, CA, USA) and following the manufacturers’ instructions in an Infinite 200Pro plate reader. Results were expressed as GPx U/mg of protein.

### SCFA analysis

Faecal samples (about 30 mg of faeces) were directly weighted to a 1.5-mL LoBind Eppendorf tube and mixed with 10 μL of internal standard mixture (BA-LAB, PA-LAB and AA-LAB) and 990 μL of a methanol:water (50:50) mixture. Samples were vortexed for 5 minutes and centrifuged (5 minutes, 15,000 rpm at 4ºC). A volume of 80 μL of the supernatant was mixed with 10 μL BHA 0.1M and 10 μL EDC 0.25M. Then, samples were vortexed and incubated at room temperature for 1 hour in darkness. After incubation, the faecal extract was diluted 20-fold in 50 per cent aqueous MeOH. 200μL of diluted sample was extracted by 600 μL of diethyl ether and 10 minutes of vigorous shaking. Then, samples were centrifuged (5 minutes, 15,000 rpm at 4ºC), and 40 μL of the upper organic layer was transferred and evaporated to dryness using an StarlettePlus-E (SPE)-dryer. The residual was reconstituted in 200 μL of 50 per cent aqueous MeOH, briefly vortexed and centrifuged (5 minutes, 15,000 rpm at 4ºC) prior to 1-μL injection on LC-MS/MS (Zeng and Cao, 2018).

The chromatographic separation was performed with a gradient, which was 0.1 per cent formic acid in water with 10 mM of ammonium formate for mobile phase A and 0.1 per cent formic acid in methanol:isopropanol (9:1 v/v) for mobile phase B. The column temperature was set at 45ºC, and the injection volume was 1 μL. The source parameters were optimised operating in positive electrospray ionisation to obtain the maximum response. The validation of the analytical methodology was carried out by analysing a faecal sample pool by standard addition, using the internal standards mentioned above. The quality parameters determined were linearity, limit of detection (MLD), limit of quantification (MQL) and both intraday and interday precision (repeatability and intermediate precision, respectively).

### Faecal DNA extraction and 16S rRNA gene amplification for microbiota composition analysis

DNA extraction was performed in fresh faecal samples collected at the end of the intervention period using the QIAamp DNA stool MiniKit according to the manufacturer´s instructions (QIAGEN, Hilden, Germany). The variable V3 and V4 regions of the bacterial 16S ribosomal RNA gene (16S rRNA) were amplified from the faecal DNA and sequenced with the Illumina MiSeq platform (2 × 300). Briefly, amplicon preparation was performed using the 16S Metagenomic Sequencing Library Preparation Protocol (Illumina, San Diego, CA, USA), which includes overhang adapter sequences for compatibility with Illumina index and sequencing adapters. Amplicon size was subsequently verified by electrophoresis (LabChip GX; PerkinElmer, Waltham, MA, USA). DNA libraries for 16S rRNA amplicons sequencing were prepared with the Nextera XT DNA Library Preparation Kit (Nextera XT; Illumina) according to the manufacturer’s instructions.

The 16S rRNA gene sequence data were processed using the Quantitative Insights Into Microbial Ecology program (QIIME 2; Bolyen et al., 2019). Low-quality reads were filtered, and chimeric sequences were removed afterwards. Clean reads were clustered as amplicon sequence variants (ASVs) using DADA2 (Callahan et al., 2016) and annotated with the SILVA v.132 16S rRNA gene reference database (Quast et al., 2013). The relative abundance of each ASV and alpha diversity (Shannon, Chao and Simpson indexes) were calculated using the phyloseq R package (McMurdie and Holmes, 2013). Weighted UniFrac distances were used to calculate Beta-diversity and then visualised with principal coordinate analysis (PCoA). Statistical significance was determined by permutational multivariate analysis of variance (PERMANOVA) with 999 random permutations using the function adonis from the vegan R package (version 2.5.7; Dixon, 2003). We performed Linear Discriminant Analysis effect size (LEfSe) to identify the bacterial taxa differentially enriched in different bacterial communities and establish microbial biomarkers (Segata et al., 2011).

### Statistical analysis

Descriptive results are presented as mean ± SEM. Statistical analyses were performed using SPSS 24.0 (SPSS, Chicago, IL, USA). In the current analysis, all variables, with the exception of microbiome variables, were normally distributed according to the Shapiro-Wilks test. Data were tested by an independent Student’s *t*-test, being *p* < 0.05 values considered as statistically significant. In the case of differences in the abundance of taxa, statistical analysis was performed with the Kruskal–Wallis test. Significance was also set up at the *p* < 0.05 level.

Correlations between differentially enriched bacterial taxa and the studied SCFAs, and phenotypic and inflammatory parameters were estimated by Spearman’s rank method, using the Microbiome package in R (available at https://microbiome.github.io/tutorials/; accessed 28 May 2021). The correlation was assessed as Coefficient ≥ 0.2 and FDR ≤ 0.05.

## Results

### Body weight, liver weight, adipose tissue weights, liver triacylglycerol content and serum parameters

**Figure 1:**
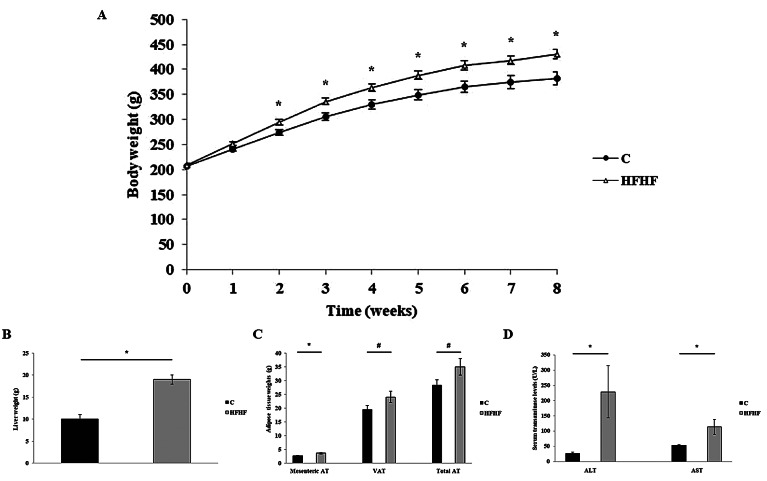
Body weight evolution (A), liver weights (B), weights of different adipose tissue depots (C) and serum transaminase levels (D) of rats fed the experimental diets for 8 weeks. Values are means ± SEM (*n* = 10). Statistical analyses were performed using unpaired Student’s *t*-test. *The level of probability was set up at *p* < 0.05 as statistically significant, and # was used to represent values of *p* < 0.1. ALT, alanine aminotransferase; AST, aspartate aminotransferase; AT, adipose tissue; C, control group; HFHF, high-fat high-fructose fed animals; VAT, visceral adipose tissue.

As shown in Figure 1A, the body weights of the animals fed the HFHF diet became significantly higher than those observed in the control group since the second week of the study and remained until the end of the experimental period (Week 8). Higher liver weights were also found in the animals of the HFHF group in comparison to the animals in the control group (Figure 1B). Indeed, this same pattern was also found regarding liver triacylglycerol content, where the values found in the HFHF group were significantly higher than those in the control group (Table 1). As far as the weight of different adipose deposits is concerned, significant differences were only found in the case of mesenteric adipose tissue. In this case, the values observed in the HFHF group were significantly higher than those found in the control group (Figure 1C). In turn, no significant changes were found between the two groups regarding visceral adipose tissue nor total adipose tissue weights, although non-statistically significant trends were found in both cases (*p* < 0.1) towards increased weights in the HFHF group.

**Table 1.** Serum glucose, insulin, non-HDL-c and MCP-1 levels, TyG index, hepatic triglyceride content, MDA content, and activities of CAT and GPx in the liver of rats fed on the experimental diets for 8 weeks.

	Control	HFHF
Serum		
Glucose (mmol/dL)	5.41 ± 0.31	6.55 ± 0.08*
Non-HDL-c	51.5 ± 3.5	108.4 ± 9.0^**^
MCP-1 (pg/mL)	210.5 ± 11.0	322.1 ± 23.7^**^
Liver		
Liver triglycerides (mg/g tissue)	17.1 ± 2.1	53.4 ± 9.5^**^
MDA (μM/mg tissue)	720.2 ± 50.5	1,008.8 ± 88.8*
CAT (nanomols/min/μg)	169.1 ± 23.1	179.6 ± 13.4
GPx (U/mg tissue)	1.26 ± 0.16	5.99 ± 1.70*

*Notes*: Values are means ± SEM (*n* = 10). Statistical analyses were performed using unpaired Student’s *t*-test.

*Abbreviations*: CAT, catalase; GPx, glutathione peroxidase; HDL-c, high-density lipoprotein cholesterol; MCP-1, monocyte chemoattractant protein-1; MDA, malondialdehyde; TyG, triglyceride-glucose index; NS, not significant.

* *p* < 0.05.

** *p* < 0.01.

With regard to serum parameters, the fasting serum glucose level observed in the animals from the HFHF group was significantly higher in comparison to that found in the control group (Table 1). Serum non-high-density lipoprotein cholesterol (non-HDL-c), which was calculated by subtracting serum high-density lipoprotein cholesterol (HDL-c) levels from total serum cholesterol levels, was significantly increased in the HFHF group compared to the control group. Finally, the analysis of serum MCP-1 levels revealed that this parameter was significantly increased in the HFHF group in comparison to the control group (Table 1).

### Hepatic oxidative stress markers

The hepatic oxidative stress analysis revealed that the amounts of MDA found in the liver samples of animals in the HFHF group were significantly greater than those found in the animals in the control group (Table 1). In addition, while no changes were observed between the two groups regarding CAT activity, a higher GPx activation was observed in the livers of the animals in the HFHF group compared to those in the control group (Table 1).

### SCFA analysis

Among all the studied SCFAs, significant differences were found in isobutyric, isovaleric and valeric acids, whose faecal concentrations were significantly greater in the HFHF group compared to the control group (Table 2).

**Table 2.** Concentration of SCFAs in faecal samples of rats fed on the experimental diets for 8 weeks.

	Control	HFHF
Acetic acid C2:0 (nmol/g)	14,234 ± 2,094	16,924 ± 2,034
Propionic acid C3:0 (nmol/g)	2,915 ± 456	5,337 ± 1,334
Isobutyric acid C4:0 (nmol/g)	449 ± 74	852 ± 125*
Butyric acid C4:0 (nmol/g)	2,126 ± 384	2,006 ± 330
Isovaleric acid C5:0 (nmol/g)	340 ± 65	870 ± 139^**^
Valeric acid C5:0 (nmol/g)	515 ± 56	1,539 ± 297*
Hexanoic acid C6:0 (nmol/g)	18 ± 4	12 ±1

*Notes*: Values are means ± SEM (*n* = 10). Statistical analyses were performed using unpaired Student’s *t*-test.

* *p* < 0.05.

** *p* < 0.01.

*Abbreviation*: NS, not significant.

### Dietary induced shifts in microbiota composition

**Figure 2:**
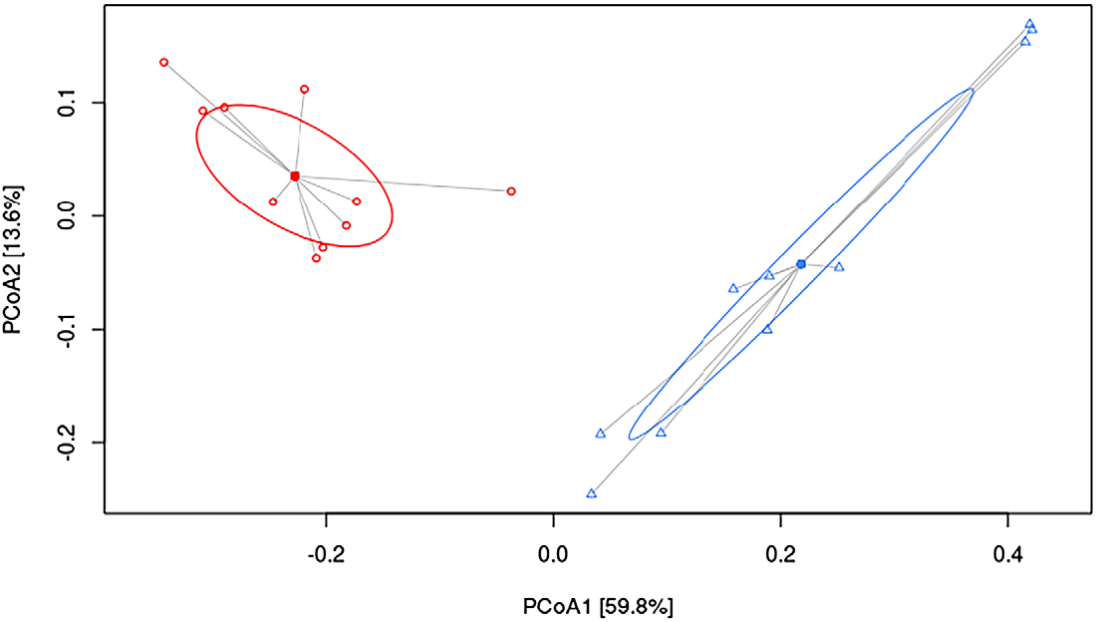
Principal coordinate analysis (PCoA) weighted UniFrac plot. Components PCoA1 and PCoA2 are shown (*p* < 0.001, PERMANOVA). All samples are connected to the centroid (shown as a point). C, control group, represented in red circles; HFHF, high-fat high-fructose fed animals, represented in blue triangles.

To understand the underlying mechanisms by which HFHF diet contributed to hepatic damage, the effects of dietary strategies on the gut microbiota composition were explored. As measured by β-diversity, there was a significant difference in overall microbial composition between the two experimental groups (*p* < 0.001, PERMANOVA). Figure 2 shows PCoA using the weighted UniFrac distance matrix. The microbiota of HFHF group was clearly separated from that of the control group.

**Figure 3:**
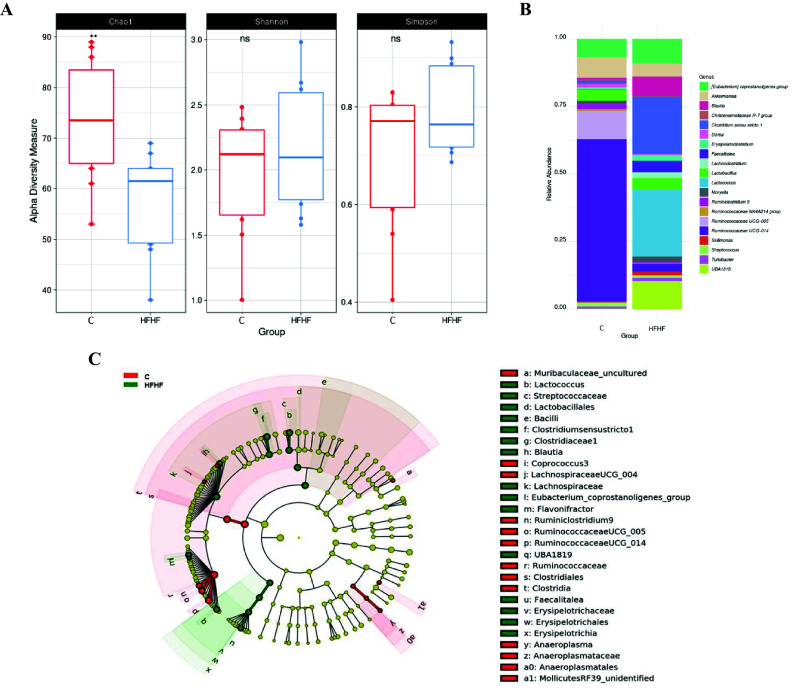
Linear discriminant analysis (LDA) integrated with effect size (LEfSe). Cladogram representing the differentially abundant taxonomic groups (LDA score > 4, *p* < 0.001) (A), microbial diversity according to Chao1, Shannon and Simpson indexes (B) and histogram representing the 20 most abundant genera (C) in rats fed on the experimental diets for 8 weeks. C, control group, represented in red; HFHF, high-fat high-fructose fed animals, represented in green.

A significant decrease in alpha diversity was observed in the HFHF group, as showed by the Chao1 index (*C* = 73.2 and HFHF = 57.2; *p* < 0.01; Figure 3A). In addition to the overall microbial composition, gut microbiota was evaluated at different levels to establish differences in the abundance of microbial taxa according to both dietary groups, and to select those bacteria which can represent potential microbial biomarkers of each condition by performing a LEfSe analysis (Figure 3B,C). In the case of the animals in the control group, the most abundant bacteria were from the Class *Clostridia*, order *Clostridiales*, family *Ruminococcaceae* (particularly the *Ruminiclostridium* 9, *Ruminococacceae* UCG 005 and *Ruminococacceae* UCG 014 genera), as well as bacteria from Class *Clostridia* (*Lachnospiraceae* UCG 004 and *Coprococcus* 3 genera) and phylum *Tenericutes* (*Anaeroplasma* and *Mollicutes* RF39 genera) were observed. Another genus of less-described bacteria (ie. *Muribaculaceae* uncultured and *Lachnospiraceae* UCG 004) was also characteristic of this group.

As far as the rats fed the HFHF diet (HFHF group) are concerned, bacteria from the phylum *Firmicutes*, class *Bacilli,* (particularly the *Lactococcus* genus), as well as bacteria from the class *Clostridia,* particularly the genera *Clostridium* sensu stricto 1, *Blautia*, *Eubacterium coprostanoligenes* group, *Flavonifractor* and the uncharacterized genus UBA1819 genera were the most abundant ones. Interestingly, these changes found in the HFHF group occurred despite at class (*Clostridia*) and order (*Clostridiales*) levels, the relative abundances in the control group were greater (Figure 3C). In addition, bacteria from the class *Erysipelotrichia* (particularly genus *Fecalitalea*) were also overrepresented in the animals from the HFHF group.

A correlation analysis looking for an association between the observed microbial biomarkers and the changes reported in phenotypic and biochemical parameters, SCFA abundances and markers of hepatic oxidative stress in the liver was conducted. A negative correlation was found between bacteria enriched in the control group fed with a standard diet (*Ruminococcaceae* UCG 005, *Ruminococcaceae* UCG 014 and *Ruminiclostridium* 9) and liver weight, transaminase (ALT) and non-HDL-c levels. On the other hand, bacteria found in HFHF group (*Clostridium sensu stricto* 1, *Clostridiaceae* 1) was correlated positively with liver weight, non-HDL-c and GPx (Table 3 and Supplementary Figure S1)

**Table 3.** Significant correlations between the relative abundance (%) of microbial biomarkers selected after LEfSe analysis A and several phenotypic and metabolic parameters in rats fed on the experimental diets for 8 weeks.

Spearman correlation (*r*) values for microbial groups founds after LEfSe analysis			
Microorganism	Liver markers	Correlation (*r*)	p.adj (FDR)
*Ruminococcaceae* UCG-014	Non-HDL-c	−0.802	0.0151
*Clostridium sensu stricto* 1	Liver weight	0.765	0.0241
*Clostridium sensu stricto* 1	Non-HDL-c	0.755	0.0278
*Clostridiaceae* 1	Liver weight	0.778	0.0347
*Clostridiaceae* 1	Non-HDL-c	0.768	0.0347
*Ruminococcaceae* UCG-014	Liver weight	−0.729	0.0387
*Ruminococcaceae* UCG-014	ALT	−0.716	0.0387
*Ruminococcaceae* UCG-005	Liver weight	−0.725	0.0388
*Ruminiclostridium* 9	Liver weight	−0.722	0.0388
*Clostridium sensu stricto* 1	GPx	0.742	0.0425

*Abbreviations:* ALT, alanine aminotransferase; GPx, glutathione peroxidase; HDL-c, high-density lipoprotein cholesterol.

## Discussion

The worldwide increase in NAFLD prevalence has converted this morbid liver condition in a global health problem, becoming the most common hepatic alteration not only in adults, but also in children (DiStefano and Shaibi, 2021; Masarone et al., 2014). NAFLD encompasses a wide spectrum of liver alterations, from a relatively benign steatosis to more harmful situations such as cirrhosis or hepatocellular carcinoma (Engin, 2017). Besides excessive lipid accumulation in the liver, events such as oxidative stress, inflammation and fibrosis are involved in the progression of hepatic damage (Brunt et al., 2015). High-fat intake, a common feature of “westernised diets,” is considered among the triggering factors of NAFLD development. In this regard, excessive dietary fat intake (specially saturated fats) can alter hepatic lipid metabolism, impairing the balance between liver lipid “input” (plasma fatty acid uptake and *de novo* lipogenesis) and “output” (mitochondrial fatty acid oxidation and very-low-density lipoprotein release), thus resulting in an excessive liver lipid accumulation (Lian et al., 2020). Additionally, much attention has also been paid to added fructose intake as another factor leading to NAFLD development. The consumption of this sugar has increased in the last decades concomitantly with the expansion of NAFLD (DiStefano and Shaibi, 2021). Indeed, excessive fructose consumption may not only increase hepatic lipid accumulation, but also produce liver inflammation and fibrosis, leading to the development of non-alcoholic steatohepatitis (Jegatheesan and De Bandt, 2017).

In accordance with these facts, in the present study, rats fed with the HFHF diet showed increased liver weight and triglyceride content, which were accompanied by an increase in the levels of serum transaminases (ALT and AST), commonly used as markers of liver function impairment (Sattar et al., 2014). Moreover, alterations in glycaemic control (higher fasting glucose) and dyslipidaemia (higher non-HDL-c levels) were also found in these same animals. These observations are in agreement with the available scientific literature, where fructose derived alterations in glucose and lipid metabolisms have been described (Softic et al., 2020; Stanhope et al., 2015).

In addition, the enhanced hepatic MDA content found in the rats fed the HFHF diet suggests that a greater ROS production occurred in these animals, which in turn resulted in higher lipid peroxidation. Moreover, the enhanced GPx activity found in the same rats points towards a higher antioxidant response, most likely as an attempt to revert the oxidative damage produced by the HFHF diet. Additionally, the higher circulating MCP-1 levels found in the HFHF group could be expected since the involvement of this cytokine in liver damage progression has been previously reported (Glass et al., 2018; Kirovski et al., 2011).

Traditionally, the so-called “two hit” theory has been used to describe the events resulting in NAFLD. According to this theory, the “first hit” is originated by insulin resistance mediated excessive hepatic lipid accumulation due to enhanced *de novo* lipogenesis and altered fatty acid transport, whereas the “second hit” accounts for hepatic oxidative stress, inflammation and mitochondrial dysfunction. All these events would lead to NAFLD development, as well as progression to nonalcoholic steatohepatitis (NASH) (Engin, 2017). However, this theory has been considered as too simplistic, and thus the “multiple hit” theory has been proposed, which, besides the aforementioned mechanisms, also considers the alterations in gut microbiota composition as one of the contributing factors (Buzzetti et al., 2016).

In the present study, the HFHF diet not only led to a decreased microbial α-diversity as revealed by lowered values in the Chao1 index, but it also affected the abundances of specific gut bacteria. Similar results were previously reported in Wistar rats fed a diet rich in fat and sucrose (45 and 17 per cent of the total energy as fat and sucrose, respectively) for 6 weeks (Etxeberria et al., 2015). Indeed, bacteria from *Ruminococcaceae* family, known to be inversely correlated with NAFLD in humans (Astbury et al., 2020), were abundant in the control group and significantly diminished in the animals fed the HFHF diet. In addition, the abundance of several bacteria genera that have been related to liver alterations, such as *Clostridium* sensu stricto 1 or *Blautia*, were increased in the HFHF group. Interestingly, these changes occurred despite at class (*Clostridia*) and order (*Clostridiales*) levels the abundances in these bacteria were greater in the control group. These findings suggest that the negative effects elicited by the HFHF feeding affects the overall gut microbiota diversity, and therefore the abundance of specific bacteria. The results obtained in this study regarding gut microbiota composition are in good accordance with those reported by other authors using rodent models fed with unbalanced experimental diets (Chen et al., 2019; Daniel et al., 2014; Duparc et al., 2017; Leal-Díaz et al., 2016). One of the limitations of this study is that the 16S rRNA analysis of gut microbiota composition was only carried out at one timepoint, using faecal samples collected at the end of the study. The main reason for not collecting faecal samples at previous time points (at the beginning of the study) is that after the adaptation period, the animals were randomly distributed in the experimental groups assuming that there were no differences between both experimental groups at baseline. Other limitation is the lack of resolution at species level of amplicon sequencing allowing characterisation of microbial changes only to genus level, and the representation of less characterised genera such as UBA1819.

Besides gut microbiota composition, the different metabolic products that are produced by intestinal bacteria have also gain interest as mediators of microbioma-host crosstalk (Bashiardes et al., 2016). Among them, SCFAs have received much attention since these metabolites are known to be involved in the maintenance of body weight and energy homeostasis or glucose and lipid metabolism, as well as being related to NAFLD development (Bashiardes et al., 2016). In this regard, SCFAs may protect against NAFLD, improving gut barrier integrity, reducing visceral adipose tissue accumulation and derived excessive fatty acid entrance into the liver, as well as by directly exerting anti-inflammatory effects once having reached the liver (Dai et al., 2020).

Acetate, propionate and butyrate are the three major volatile SCFAs produced by gut bacteria and are known to participate in a variety of processes (Deleu et al., 2021). In the case of the liver, studies conducted in rodents and humans have described hepato-protective effects for these SCFAs. Several mechanisms of action have been described to date regarding the effects of SCFAs in liver protection (Dangana et al., 2020) including the modulation of visceral adipose tissue fat accumulation and lipid metabolism. Moreover, the improvement produced by these SCFAs in gut barrier function is also potentially responsible for their hepato-protective effects. Additionally, the activation of nod-like receptor family pyrin domain containing 3 inflammasome and resulting release of interleukin 18 produced by acetate, butyrate and propionate has also been described to improve gut barrier integrity (Macia et al., 2015). Despite the well characterised hepato-protective effects of acetate, propionate and butyrate, no differences on their levels were found between the control and HFHF groups. As far as the enhanced faecal level of isobutyric acid found in the HFHF group of our study, it could be considered as expected since increased faecal level of this bacterial product has been found in patients with NAFLD (Da Silva et al., 2018; Jumpertz et al., 2011). In addition, a greater level of isobutyric acid has been reported in faecal samples of subjects with hypercholesterolemia in the study reported by Granado-Serrano et al. (2019), where a positive correlation between faecal isobutyric acid level and serum low-density lipoprotein cholesterol level was observed. Moreover, in that study, a higher faecal isovaleric acid concentration was also found in patients with hypercholesterolemia, which fits well with the results of our study, where the rats fed the HFHF diet showed the same effect (Granado-Serrano et al., 2019). However, in our case, no correlation was found between the level of this SCFA and non-HDL-c-levels. As valeric acid is concerned, the studies addressing the effects of this SCFA in liver steatosis are scarce.

In order to better understand the potential associations between the studied microbial measurements and the phenotypical, biochemical and SCFA levels, correlation studies were carried out. According to the results obtained, bacteria from the *Ruminococcaceae* family could be considered as markers of low liver weight and/or serum transaminase and non-HDL-c levels, since negative correlations were found in the control group between these bacteria and the aforementioned markers. These results could be explained because these bacteria have been related to gastrointestinal health in humans due to their effect in the maintenance of intestinal structure and functions, such as permeability, nutrient uptake and immunocompetence (Rajilić‐Stojanović and de Vos, 2014; Tang et al., 2018). As far as the positive correlations found in the HFHF group between *Clostridium sensu stricto* 1 and *Clostridiaceae* 1 with parameters such as non-HDL-c and GPx, these results are in line with data reported in studies in humans, in which the abundance of these bacteria was positively correlated with HDL-c molecule diameter (Vojinovic et al., 2019). However, other studies have also reported negative correlations of these bacteria with markers such as liver stiffness measurement and controlled attenuation parameter in humans (Lanthier et al., 2021). Indeed, reductions in abundance have been related to the development of liver fibrosis in patients with severe steatosis (Lanthier et al., 2021). Therefore, despite *Clostridium sensu stricto* 1 and *Clostridiaceae* 1 are indicators of less healthy microbiota (Yang et al., 2019), further studies are warranted in order to better understand this apparent discrepancy. With regard to SCFAs, despite significant differences in their levels were found between the control and HFHF groups, no correlations were found with gut microbiota composition. These outcomes may result unexpected, especially for rats in the control group whose faeces revealed a greater presence of butyrate-producing bacteria such as *Ruminococcaceae,* but without an actual change of the levels of butyrate. Similarly, increased acetate levels could also been expected in the animals fed the HFHF diet since a relationship between this SCFA and metabolic syndrome has been proposed through a microbiota-brain axis (Perry et al., 2016). In this scenario, it could be hypothesised that the observed changes in gut microbiota composition were not big enough to shift the production of certain SCFA. In this regard, a longer experimental period could have resulted in a clearer relationship between shifts in gut microbiota composition and SCFA levels.

In conclusion, the current study demonstrates that the alterations induced by a high-energy diet rich in saturated fat and fructose in the liver, as well as the impairment of several metabolic markers involved in glycaemic control and lipid homeostasis, may be related, at least in part, to changes in overall gut microbiota composition and the abundance of specific bacteria derived by this dietary pattern since several mechanisms of action have been characterised (increased gut permeability, enhanced bacterial translocation and production/release of pro-inflammatory mediators or inflammation of adipose tissue and liver) linking the two events (Deleu et al., 2021). Nevertheless, further determinations are warranted to better elucidate/confirm this hypothesis. As far as SCFA is concerned, the differences found between the experimental groups were not extensive despite the significant changes induced by the HFHF diet in gut microbiota richness and diversity. It seems that under these experimental conditions, the role played by SCFAs in diet-induced steatohepatitis development seems limited and may not be associated with gut microbiota shifts.

## Supplementary material

For supplementary material accompanying this paper visit https://doi.org/10.1017/gmb.2022.2. click here to view supplementary material
