# Supplementary figures and images for: Involvement of microbiota and short-chain fatty acids on non-alcoholic steatohepatitis when induced by feeding a hypercaloric diet rich in saturated fat and fructose
Source: Gut Microbiome (Camb). 2022 Apr 8;3:e5. doi: 10.1017/gmb.2022.2 (PMC11406367; doi:10.1017/gmb.2022.2)

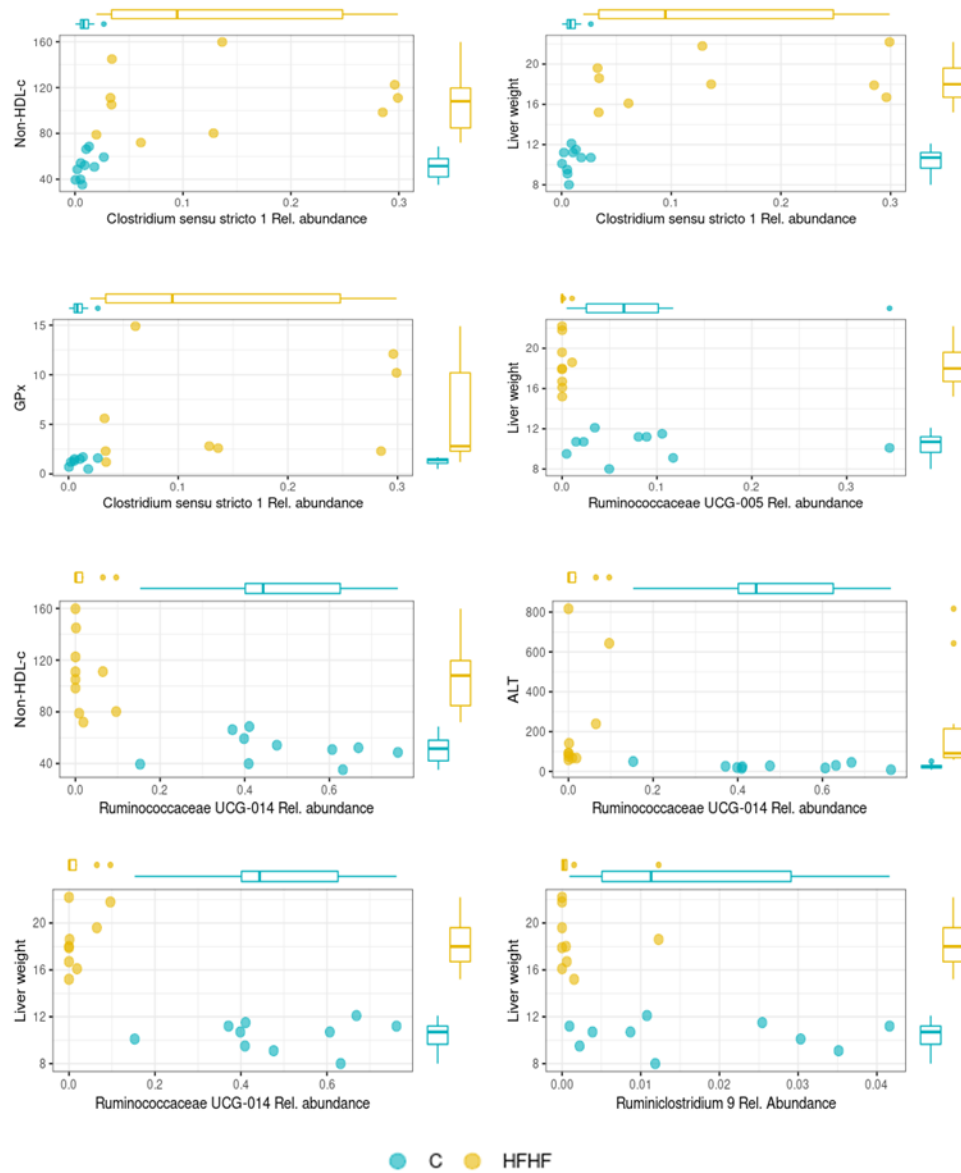

Supplement: Supplementary file 1 [file S2632289722000020sup001.pdf]
